# Spatial heterogeneity of lineage-committed muscle stem cell subpopulations challenges bipotency assumptions in Thai-French crossbred cattle

**DOI:** 10.14202/vetworld.2026.4025-4041

**Published:** 2026-09-14

**Authors:** Saowaluk Saisomboon, Kulthida Vaeteewoottacharn, Nutsara Settha, Jatuporn Pattanachot, Raynoo Thanan, Vichai Leelavatcharamas, Kantiya Petsong, Rina Patramanon, Araya Chaoruangrit, Thidarut Boonmars

**Affiliations:** 1 Department of Biochemistry, Faculty of Medicine, Khon Kaen University, Khon Kaen 40002, Thailand.; 2 Department of Biotechnology, Faculty of Technology, Khon Kaen University, Khon Kaen 40002, Thailand.; 3 Department of Food Technology, Faculty of Technology, Khon Kaen University, Khon Kaen 40002, Thailand.; 4 Department of Biochemistry, Faculty of Science, Khon Kaen University, Khon Kaen 40002, Thailand.; 5 Department of Parasitology, Faculty of Medicine, Khon Kaen University, Khon Kaen 40002, Thailand.

**Keywords:** bovine muscle stem cells, cultured meat, fibro-adipogenic progenitors, lineage commitment, satellite cells, spatial heterogeneity, stem cell isolation, Thai-French crossbred cattle

## Abstract

**Background and Aim:**

Muscle-derived stem cells (MDSCs) are promising cellular resources for cultured meat because skeletal muscle contains progenitors that can generate myogenic and adipogenic tissues. However, whether the apparent dual-lineage differentiation of CD29-positive MDSCs reflects intrinsic bipotency or heterogeneous, lineage-committed subpopulations remains unclear. This study aimed to establish a simplified protocol for isolating CD29-positive MDSCs from adult Thai-French crossbred cattle, characterize satellite cell (SC) and fibro-adipogenic progenitor (FAP) subpopulations, and determine whether their spatial composition governs differentiation outcomes.

**Materials and Methods:**

Skeletal muscle samples were obtained from three adult male Pon Yang Kham Thai-French crossbred cattle. Five independent tissue samples were used to validate cell isolation, while three spatial isolates (BNS1–BNS3) from distinct chuck roll locations of one donor were used for detailed characterization. Muscle-derived cells were isolated by enzymatic digestion, filtration, red blood cell lysis, and short-term culture enrichment. CD29-positive cells were characterized and separated into CD56-positive SCs and CD56-negative FAPs using fluorescence-activated cell sorting. Proliferation, immunofluorescence, lipid accumulation, and quantitative gene expression analyses were performed to assess lineage-specific differentiation.

**Results:**

The protocol yielded 5.13 ± 1.23 × 10⁵ viable cells/g tissue and >95% CD29-positive enrichment. Spatial isolates showed pronounced variation in SC:FAP composition, ranging from 4.3:95.1 to 96.0:3.8. Both subpopulations exhibited approximately 6–8-fold proliferative capacity during early passages. SCs formed multinucleated myotubes and expressed *Myf5*, *MyHC*, and *MyoG* following myogenic induction. FAPs accumulated intracellular lipids and showed increased *CIDEC* expression following adipogenic induction. Unfractionated CD29-positive populations displayed reciprocal lineage-directed responses under myogenic and adipogenic conditions, indicating that differentiation behavior was determined by the composition and selective induction of pre-existing lineage-biased subpopulations.

**Conclusion:**

CD29-positive MDSCs from adult Thai-French crossbred cattle represent spatially heterogeneous mixtures of lineage-committed SCs and FAPs rather than a uniformly bipotent progenitor population. The simplified isolation approach provides an accessible source of expandable bovine progenitor cells, while the marked spatial heterogeneity highlights the need for subpopulation-aware cell sourcing and quality control to improve reproducibility in cultured meat production.

## INTRODUCTION

Skeletal muscle is a highly dynamic tissue with remarkable regenerative capacity that depends on resident stem and progenitor cell populations to maintain tissue homeostasis and repair damage throughout life [1–3]. Among these populations, satellite cells (SCs), which reside beneath the basal lamina of myofibers, serve as the principal myogenic progenitors responsible for postnatal skeletal muscle growth, maintenance, and regeneration [2, 4, 5]. In parallel, the muscle interstitium contains fibro-adipogenic progenitor cells (FAPs), a distinct stromal population capable of adipogenic differentiation and contributing to intramuscular fat deposition under appropriate conditions [6–8]. The coordinated activities of SCs and FAPs influence skeletal muscle regeneration, composition, and function. In livestock, the balance between myofiber formation and intramuscular adipose tissue deposition is particularly important because it contributes to economically relevant meat quality characteristics, including marbling [9–11].

The increasing global demand for dietary protein, particularly in rapidly developing regions such as East and South Asia, has intensified interest in sustainable and scalable alternatives to conventional livestock production [12]. Cultured meat, defined as edible muscle tissue produced *in vitro* from animal-derived cells, has emerged as a potential approach for addressing challenges associated with the environmental impact of livestock production, animal welfare, and food security [13–16]. In this context, Thailand and other Association of Southeast Asian Nations countries have increasingly emphasized “Future Food” initiatives, creating opportunities to develop advanced alternative-protein technologies [17]. Producing structured cultured meat that reproduces key characteristics of conventional beef requires both myogenic and adipogenic components; therefore, coordinated incorporation of progenitor populations capable of generating muscle and adipose tissues is important for product development [6, 18].

Researchers have investigated several cellular sources for cultured meat production. Pluripotent stem cells, including embryonic stem cells and induced pluripotent stem cells (iPSCs), offer extensive differentiation potential but raise ethical, safety, and regulatory concerns that may limit their use [19–22]. In contrast, muscle-derived stem cells (MDSCs) provide biologically relevant progenitor populations from a single tissue source and are therefore practical candidates for cultured meat production [4]. Researchers have developed several approaches to isolate and enrich MDSCs from skeletal muscle, including pre-plating, density-gradient centrifugation, and fluorescence-activated cell sorting (FACS) [23]. Among available cell-surface markers, cluster of differentiation 29 (CD29), the β1 integrin subunit, has been widely used to identify and enrich bovine and porcine MDSC populations [9, 24]. CD29-positive populations have been reported to contain CD56-positive SCs and CD56-negative FAPs and to exhibit both myogenic and adipogenic differentiation *in vitro* [9, 25]. These observations have contributed to the interpretation of CD29-positive MDSCs as a bipotent progenitor population.

However, an important biological uncertainty remains regarding the cellular basis of this apparent bipotency. CD29-positive fractions are heterogeneous, and their relative proportions of SCs and FAPs can vary according to tissue source, spatial origin, and isolation conditions [9, 26]. Consequently, demonstration of myogenic and adipogenic differentiation at the population level does not necessarily establish that individual CD29-positive cells possess intrinsic bipotency. Instead, the observed dual-lineage differentiation could result from the presence and selective expansion or induction of pre-existing lineage-committed subpopulations within the heterogeneous CD29-positive fraction. Distinguishing between these mechanisms is important because population level differentiation assays may otherwise lead to an overestimation of cellular multipotency. Furthermore, variation in the baseline proportions of SCs and FAPs could contribute to inconsistent differentiation outcomes among tissue isolates, with direct implications for reproducibility and standardization in cultured meat production.

A further knowledge gap concerns the applicability of existing MDSC isolation approaches to adult livestock tissues obtained under practical production conditions. Previous approaches have often relied on specialized enrichment procedures, whereas scalable cultured meat applications require accessible, reproducible methods applicable to adult tissues from routine livestock production systems. In addition, MDSCs from Southeast Asian crossbred cattle remain uncharacterized, leaving uncertainty about their cellular composition, proliferative behavior, and lineage-specific differentiation potential. Pon Yang Kham Thai-French crossbred cattle represent a geographical indication (GI)-registered premium cattle population of economic importance in Thailand [27, 28]. Characterizing MDSCs from this locally relevant genetic resource may therefore provide both biological information on bovine muscle progenitor heterogeneity and a practical cellular foundation for regionally relevant cultured meat research.

Therefore, this study aimed to develop and validate a simplified, reproducible protocol to isolate and expand CD29-positive MDSCs from adult Pon Yang Kham Thai-French crossbred cattle using slaughterhouse-derived skeletal muscle tissue. The protocol incorporated enzymatic digestion, size-based filtration, and short-term culture enrichment to facilitate cell recovery under practical conditions. After expansion, we characterized CD29-positive populations by cell-surface marker expression and separated them by FACS into CD56-positive SCs and CD56-negative FAPs. Their proliferative capacity and lineage-specific differentiation potential were subsequently evaluated, together with the differentiation behavior of unfractionated CD29-positive populations under myogenic and adipogenic induction conditions. Specifically, the study sought to (1) establish a practical MDSC isolation protocol applicable to adult livestock tissue obtained under field conditions; (2) characterize the composition, proliferative behavior, and differentiation capacity of CD29-positive bovine MDSC subpopulations; and (3) determine whether the apparent dual-lineage differentiation of CD29-positive MDSCs is better explained by intrinsic bipotency or by the relative composition and lineage-directed responses of pre-existing SC and FAP subpopulations.

## MATERIALS AND METHODS

### Ethical approval

The study protocol and all procedures involving the collection and use of bovine skeletal muscle tissue were reviewed and approved by the Institutional Animal Care and Use Committee of the Faculty of Medicine, Khon Kaen University, Thailand (approval no. AEMDKKKU 014/2022). Skeletal muscle tissues were obtained from adult male Pon Yang Kham Thai-French crossbred cattle (*Bos taurus*) slaughtered at the Pon Yang Kham Livestock Breeding Cooperative, Sakon Nakhon, Thailand, as part of routine commercial slaughter; no animals were slaughtered specifically for the purposes of this study. Tissue collection and handling were conducted in accordance with the Good Manufacturing Practice standards of the Pon Yang Kham Livestock Breeding Cooperative Limited. The collected tissues were then used to isolate, expand, phenotypically characterize, and differentiate MDSCs *in vitro*. All applicable experimental procedures were conducted in accordance with the approved institutional protocol, and the study was reported in accordance with the ARRIVE 2.0 guidelines, where applicable.

### Study period and location

The study was conducted from December 2022 to July 2023. Skeletal muscle samples were collected from adult male Pon Yang Kham Thai-French crossbred cattle slaughtered at the Pon Yang Kham Livestock Breeding Cooperative, Sakon Nakhon, Thailand [27, 28]. Subsequent isolation, culture, characterization, and experimental analyses of muscle-derived cells were performed at the Department of Biochemistry, Faculty of Medicine, Khon Kaen University, Thailand.

### Study design

This experimental study was designed to develop and validate a simplified protocol for isolating and expanding CD29-positive MDSCs from adult Thai-French crossbred cattle and to characterize the spatial heterogeneity and lineage-specific differentiation of their constituent SC and FAP populations. Skeletal muscle tissues from three donor animals were used for protocol validation and assessment of viable cell yield and CD29-positive enrichment efficiency. Detailed subpopulation characterization and functional assays were subsequently conducted using three independent spatial isolates (BNS1, BNS2, and BNS3) obtained from distinct locations within the chuck roll of a single donor animal. Thus, BNS1–BNS3 represented within-animal spatial replicates rather than independent biological donors. The experimental workflow comprised tissue collection and processing, MDSC isolation and expansion, flow cytometric characterization and FACS-based separation of SCs and FAPs, proliferation assays, lineage-specific differentiation, gene expression analysis, immunofluorescence staining, and mycoplasma contamination testing.

### Tissue collection from Thai-French cattle

Skeletal muscle tissue was collected from three adult male Pon Yang Kham Thai-French crossbred cattle (*Bos taurus*) (N = 3 animals; age, 2.5–3 years; body weight, 532–650 kg) slaughtered at the Pon Yang Kham Livestock Breeding Cooperative, Sakon Nakhon, Thailand [27, 28]. Tissue samples from all three animals were used to validate the isolation protocol and determine viable cell yield per gram of tissue and CD29-positive enrichment efficiency. For detailed subpopulation characterization, differentiation assays, and SC:FAP heterogeneity analysis, three independent spatial isolates (BNS1, BNS2, and BNS3) were obtained from three distinct locations within the chuck roll of a single donor (Animal 3; N = 1 animal; n = 3 spatial isolates; age, 34 months; body weight, 532 kg; slaughtered on July 11, 2023). Therefore, BNS1–BNS3 represented within-animal spatial replicates and not independent animals.

Muscle tissue was obtained from the chuck roll, a commercial designation for a boneless forequarter subprimal (IMPS/NAMP item 116A) comprising *Musculus longissimus dorsi*, *Musculus rhomboideus*, *Musculus spinalis dorsi*, *Musculus complexus*, *Musculus multifidus dorsi*, *Musculus serratus ventralis*, *Musculus subscapularis*, and *Musculus splenius*. Tissue blocks were excised from three distinct anatomical locations within this subprimal. The subprimal was not seam-dissected into individual muscles at the time of collection; therefore, the specific muscle of origin of each tissue block was not recorded.

At the slaughterhouse, tissue samples were placed in sterile 50-mL centrifuge tubes, rinsed once with 75% ethanol and twice with sterile normal saline solution (NSS) with vigorous shaking, and cut into 0.1–0.2 cm³ pieces. The samples were maintained in ice-cold NSS until further processing or cryopreservation. For cryopreservation, dissected tissue samples were stored in fetal bovine serum (FBS) containing 10% dimethyl sulfoxide (DMSO) at −80°C.

### Isolation and expansion of muscle-derived cells

Muscle-derived cells were isolated using a modified previously described protocol [6]. Tissue fragments were enzymatically dissociated in 0.2% collagenase type I (C0130, ≥125 CDU/mg solid; Sigma-Aldrich, St. Louis, MO, USA) prepared in serum-free Dulbecco's modified Eagle medium with high glucose (DMEM-HG; 25 mM glucose; Gibco BRL, Carlsbad, CA, USA). Samples were incubated at 37°C for 1.5 h under continuous rotation at 20 rpm using a MACSmix™ Tube Rotator (Miltenyi Biotec, Bergisch Gladbach, Germany).

The cells were washed once with DMEM supplemented with 20% FBS (20% FBS-DMEM-HG) and filtered through a 200-µm nylon mesh to remove undigested tissue debris. The resulting cell pellet was treated with red blood cell (RBC) lysis buffer containing 15 mM NH₄Cl, 1 mM KHCO₃, and 10 µM ethylenediaminetetraacetic acid (EDTA) for 5 min and subsequently washed twice with phosphate-buffered saline (pH 7.4). Viable cells were quantified using the trypan blue exclusion method (Gibco BRL).

Isolated cells were seeded at an initial density of 1.8–3.8 × 10⁵ cells/well in 6-well plates and cultured in basic MDSC culture medium (BMCM), consisting of 20% FBS-DMEM-HG supplemented with 5 ng/mL basic fibroblast growth factor (bFGF; R&D Systems, Minneapolis, MN, USA) and 2.5 µM p38 mitogen-activated protein kinase (MAPK) inhibitor SB203580 (Selleck Chemicals, Houston, TX, USA) to support proliferation and delay spontaneous differentiation [29]. Cells were passaged at approximately 60% confluence for subsequent experiments.

### Detection of cell-surface marker expression and purification of SCs and FAPs

Cell-surface marker expression was evaluated using allophycocyanin (APC)-conjugated anti-human CD29 (clone TS2/16; BioLegend, San Diego, CA, USA), fluorescein isothiocyanate (FITC)-conjugated anti-sheep CD31 (clone CO.3E1D4; Bio-Rad, Hercules, CA, USA), FITC-conjugated anti-sheep CD45 (clone 1.11.32; Bio-Rad), and phycoerythrin (PE)-conjugated anti-human CD56 (clone MEM-188; BioLegend). Cells were expanded for 28–48 days before FACS.

Flow cytometry was performed using a FACSCanto™ II system (BD Biosciences, Franklin Lakes, NJ, USA), and the resulting data were analyzed using FlowJo™ software version 10.4 (Tree Star, Ashland, OR, USA). CD29⁺/CD56⁺ SCs and CD29⁺/CD56⁻ FAPs were isolated by FACS using a BD FACSAria™ III Cell Sorter (BD Biosciences). The approximate cell inputs and sorted yields were as follows: BNS1, approximately 7 × 10⁵ input cells, 1 × 10⁴ sorted FAPs, and 1.4 × 10⁵ sorted SCs; BNS2, approximately 1.4 × 10⁶ input cells, 4.7 × 10⁵ sorted FAPs, and 2.4 × 10⁵ sorted SCs; and BNS3, approximately 7 × 10⁵ input cells, 9.3 × 10³ sorted FAPs, and 4.1 × 10⁵ sorted SCs.

Sorted SCs were maintained in BMCM, whereas FAPs were cultured in minimum essential medium alpha (MEM-α; Gibco BRL) supplemented with 15% FBS (15% FBS-MEM-α). Both cell types were cultured with 1× antibiotic-antimycotic at 37°C in a humidified atmosphere containing 5% CO₂.

### Cell proliferation assay

All functional assays, including proliferation and myogenic and adipogenic differentiation, were performed using early-passage cells (P2–P8 post-sorting). Proliferative capacity was evaluated using a colorimetric cell counting assay. SCs and FAPs were seeded at 3 × 10³ cells/well in 96-well plates and cultured for 24, 48, and 72 h. At each time point, SuperKine™ Maximum Sensitivity Cell Counting Kit-8 (CCK-8; Boster Biological Technology, Atlanta, GA, USA) solution was added, and the cells were incubated for 2 h at 37°C. Absorbance at 450 nm (OD₄₅₀) was measured using an EZ Read 2000 microplate reader (Biochrom, Cambridge, UK), and the data were analyzed using Galapagos software (Biochrom). Baseline OD₄₅₀ was recorded 6 h after plating and designated as day 0. Proliferative capacity was expressed as the fold change relative to the day 0 value.

### Myogenic differentiation

Myogenic differentiation of SCs was performed using a protocol adapted from a previously described method [29]. SCs were seeded on plates coated with 50 µg/mL rat-tail collagen type I (A10483-01, lot no. 531799; Invitrogen, Carlsbad, CA, USA) in 20% FBS-DMEM-HG and cultured for 24 h to promote attachment. The cells were subsequently transferred to myogenic differentiation medium (MDM), consisting of DMEM with low glucose (DMEM-LG; 5.6 mM glucose) supplemented with 2% FBS and 1 µg/mL insulin (I6634; Sigma-Aldrich). The medium was replaced every 2 days, and the cells were harvested on day 7 for analysis.

### Adipogenic differentiation

Adipogenic differentiation of FAPs was performed using a method adapted from Dohmen *et al.* [6]. FAPs were pre-cultured overnight in 15% FBS-MEM-α and subsequently transferred to adipogenic differentiation medium (ADM), consisting of 1% FBS-MEM-α supplemented with 1 µg/mL insulin. During the first 3 days, ADM was supplemented with three adipogenic inducers: 0.5 mM 3-isobutyl-1-methylxanthine (IBMX; I5879), 100 µM indomethacin (I7378), and 1 µM dexamethasone (D1756) (all from Sigma-Aldrich). The medium was replaced every 2 days, and the cells were analyzed on day 14.

For lineage-specific differentiation of unfractionated CD29-positive MDSCs, a protocol adapted from Naraoka *et al.* [9] was used. Cells were pre-cultured for 3 days in BMCM on collagen-coated or uncoated plates. Myogenic differentiation was induced by culturing the cells in MDM-2 (2% FBS-DMEM-HG supplemented with 1 µg/mL insulin) for 7 days. For adipogenic differentiation, cells were sequentially cultured in MDM-2 for 7 days followed by ADM for 14 days.

### RNA extraction and real-time reverse transcription–polymerase chain reaction

Total RNA was extracted using TRIzol™ reagent (Invitrogen) according to the manufacturer's protocol. RNA (0.5 µg) was reverse-transcribed using ReverTra Ace™ qPCR RT Master Mix with gDNA Remover (Toyobo, Osaka, Japan). Quantitative real-time polymerase chain reaction (qPCR) was performed using a LightCycler® 480 system (Roche Diagnostics, Mannheim, Germany) with 100 ng complementary DNA (cDNA), 1 µM primers, and SYBR Green I Master Mix (Roche). Melting-curve analysis was performed to confirm qPCR product specificity. *GAPDH* served as the internal reference gene.

For each cell isolate and experimental condition, RNA was extracted from three independent culture replicates (Batches 1–3), and each RNA sample was analyzed in technical duplicate. Only amplification curves with crossing point (Cp) values before cycle 35 were considered detectable. Samples showing no amplification or amplification at or after cycle 35 were classified as undetectable and excluded from relative expression calculations. Genes for which fewer than three batches yielded detectable amplification are identified in the corresponding figure legends, together with the number of contributing batches. Relative gene expression was calculated using the 2^−ΔCp method, where ΔCp = Cp_target − Cp_*GAPDH*. Primer sequences are presented in Supplementary Table 1 [6, 29].

### Immunofluorescence staining

Cells were fixed with 4% paraformaldehyde for 15 min, permeabilized with 0.2% Triton X-100 for 10 min, and blocked with 5% FBS for 1 h at room temperature. For detection of the myogenic marker, cells were incubated overnight at 4°C with mouse anti-myosin heavy chain antibody (1:500; MY-32; Sigma-Aldrich), followed by Alexa Fluor™ 647-conjugated goat anti-mouse immunoglobulin G secondary antibody (1:500; Invitrogen). Nuclei were counterstained with Hoechst 33342 (Thermo Fisher Scientific, Waltham, MA, USA).

For visualization of intracellular lipids, cells were stained with 2 µM BODIPY™ 493/503 (Sigma-Aldrich) at 37°C for 15 min in the dark, fixed with 4% paraformaldehyde, and counterstained with Hoechst 33342. Fluorescence images were acquired using a ZEISS LSM 710 confocal microscope with ZEN software (Carl Zeiss Microscopy GmbH, Jena, Germany).

### Mycoplasma contamination testing

All cell cultures were evaluated for mycoplasma contamination using the MycoAlert® Mycoplasma Detection Kit (LT07-318; Lonza, Switzerland) according to the manufacturer's enzymatic protocol. All tested cultures were negative for mycoplasma contamination.

### Statistical analysis

Three biological isolates (BNS1, BNS2, and BNS3) derived from a single donor animal (Animal 3) were used to assess spatial/intra-donor heterogeneity. For gene expression analyses, each isolate and experimental condition comprised three independent culture batches (Batches 1–3), with each batch analyzed in technical duplicate. The mean of the technical duplicates was calculated for each batch, and the independent batches served as the units of statistical analysis.

Data are presented as the mean ± standard deviation. Normality was assumed based on the experimental design and small number of replicates. Differences between experimental conditions were evaluated using Student's t-test for pairwise comparisons and one-way analysis of variance (ANOVA) for multiple-group comparisons. p < 0.05 was considered statistically significant. No correction for multiple comparisons was applied because each gene was analyzed as an independent biological marker rather than as part of a family-wise hypothesis. All statistical analyses were performed using GraphPad Prism version 10 (GraphPad Software, Boston, MA, USA).

## RESULTS

### Isolation of CD29-positive MDSCs

**Muscle-derived cell isolation and expansion:** Muscle tissue from the chuck roll of adult Pon Yang Kham Thai-French crossbred cattle was dissected into fragments with an average weight of 58.2 ± 4.3 mg and subjected to enzymatic digestion with collagenase, followed by size-based filtration, RBC lysis, and washing. The isolation protocol consistently yielded 5.13 ± 1.23 × 10⁵ viable cells/g of tissue, with cell viability exceeding 80% across all isolates (Supplementary Table 2). During the pre-sorting expansion phase, cells proliferated 3–6-fold within 10 days under BMCM conditions and were subcultured once to twice per week. Three to four passages were sufficient to obtain the cell numbers required for flow cytometric analysis and FACS-based sorting.

To preserve tissue resources, dissected muscle samples were cryopreserved in FBS containing 10% DMSO at −80°C. After thawing, the cryopreserved tissues remained viable and yielded muscle-derived cell numbers comparable to those from freshly processed tissues, indicating the feasibility of tissue cryopreservation for subsequent experiments. Long-term cryopreservation stability beyond 100 days was not evaluated in this study.

**Subpopulation identification and FACS-based isolation of SCs and FAPs:** CD29-positive MDSCs were characterized by flow cytometry using a panel of cell-surface markers. Following pre-sorting culture, >95% of the cells expressed CD29 across all isolates, whereas CD31- and CD45-positive cells were not detected, indicating effective depletion of endothelial and hematopoietic cell populations (Supplementary Figure 1). Within the CD29-positive population, two major subpopulations were distinguished according to CD56 expression: CD29⁺/CD56⁺ SCs and CD29⁺/CD56⁻ FAPs [9, 29].

The cell isolation and sorting workflow is summarized in Figure 1A. The relative proportions of SCs and FAPs varied markedly among the tissue isolates (Figure 1B). The SC:FAP ratios ranged from 4.3:95.1 in the FAP-dominant BNS1 isolate to 96.0:3.8 in the SC-enriched BNS3 isolate, with the remaining cells representing CD29-negative populations. These findings demonstrated pronounced spatial variation in SC:FAP composition among isolates obtained from a single bovine donor.

Following flow cytometric characterization, SCs and FAPs were purified by FACS using a conservative gating strategy that excluded boundary events between the CD29⁺/CD56⁺ and CD29⁺/CD56⁻ gates to minimize cross-contamination between the two subpopulations. Consequently, the percentages of cells recovered in the sorted fractions were slightly lower than their corresponding proportions in the pre-sorting populations. BNS1 yielded a predominantly FAP fraction, accounting for 88.5% of the pre-sorting population. In contrast, BNS3 yielded a predominantly SC-enriched fraction (85.9%). BNS2 contained both subpopulations, comprising 68.4% FAPs and 21.6% SCs, enabling comparative analyses of the two cell types derived from the same tissue isolate (Figure 1C). No apparent morphological differences were observed between sorted SCs and FAPs by phase-contrast microscopy (Figure 1D). Representative FACS gating plots are presented in Supplementary Figures 2–4.

### Characterization of SCs and FAPs

**Proliferative capacity:** The proliferative capacity of SCs and FAPs was evaluated across multiple passages using the CCK-8 assay. Both cell types proliferated robustly during early passages, with cell yield increasing approximately 6–8-fold over the first four passages (passages 7–8 after initial isolation). Proliferative capacity subsequently declined with increasing passage number, and cell division was rarely observed at passages 15–16 (Figures 2A–D).

The PDT of BNS1-derived FAPs increased from 21.8 h at passage 4 to 27.0 h at passage 6 and 29.8 h at passage 8. For BNS2-derived FAPs, the corresponding PDTs were 23.6, 32.7, and 39.1 h, whereas BNS2-derived SCs showed PDTs of 19.8, 24.9, and 25.5 h at passages 4, 6, and 8, respectively. BNS3-derived SCs showed PDTs of 21.1 h at passage 4, 23.1 h at passage 6, and 23.6 h at passage 8. Thus, a passage-dependent increase in PDT and a decline in proliferative capacity were observed across the evaluated isolates and cell types, consistent with previously reported proliferative limitations of primary adult muscle stem cells during prolonged serum-dependent culture [30–32].

**Figure 1:**
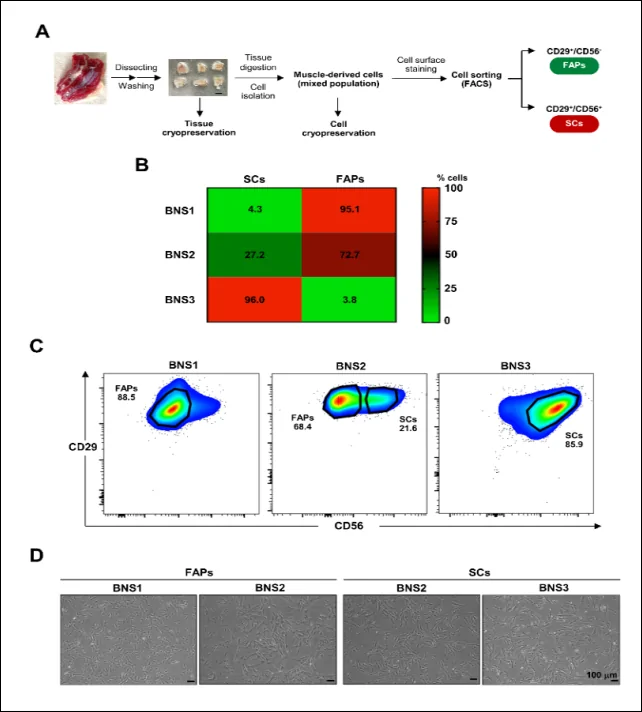
Isolation and subpopulation profiling of CD29-positive muscle-derived stem cells from Pon Yang Kham Thai-French crossbred cattle. (A) Schematic workflow for the isolation and enrichment of CD29-positive MDSCs from chuck roll skeletal muscle tissue. Scale bar = 0.5 cm. (B) Heatmap showing the mean percentages of SC and FAP populations in the pre-sorted isolates. (C) Representative FACS plots showing the gating strategy used to identify and sort FAPs (CD29⁺/CD56⁻) and SCs (CD29⁺/CD56⁺) from BNS1, BNS2, and BNS3. Black lines indicate selection boundaries, and percentages indicate the proportions of each subpopulation within the CD29-positive gate. Pronounced spatial variation in the SC:FAP ratio was evident among isolates. (D) Representative phase-contrast micrographs of sorted SCs and FAPs at equivalent passages. No apparent morphological differences were observed between the subpopulations. Images were captured at 4× objective magnification. Scale bar = 100 µm. FACS = Fluorescence-activated cell sorting; FAPs = Fibro-adipogenic progenitor cells; MDSCs = Muscle-derived stem cells; SCs = Satellite cells.

**Figure 2:**
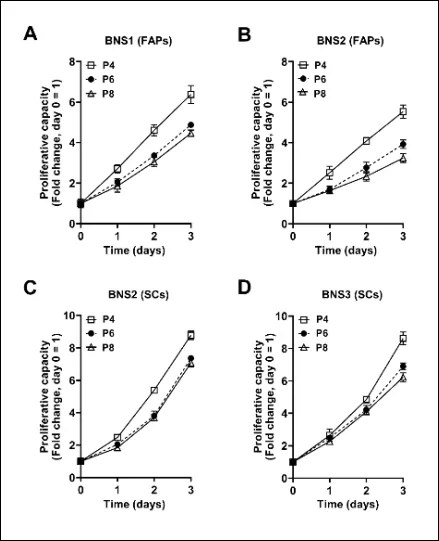
Proliferative capacity of CD29-positive muscle-derived stem cell subpopulations across passages. Fold change in cell number relative to day 0, measured by OD₄₅₀ using the CCK-8 assay, for FAPs from (A) BNS1 and (B) BNS2 and SCs from (C) BNS2 and (D) BNS3. All evaluated subpopulations showed robust proliferation during the early passages, followed by a gradual decline at later passages. Data are presented as mean ± standard deviation from three independent experiments. CCK-8 = Cell Counting Kit-8; FAPs = Fibro-adipogenic progenitor cells; OD₄₅₀ = Optical density at 450 nm; SCs = Satellite cells.

**Adipogenic differentiation of FAPs:** FAPs derived from BNS1 and BNS2 were cultured in ADM for 14 days to evaluate their adipogenic differentiation capacity. Both isolates showed increased intracellular lipid accumulation following adipogenic induction, as demonstrated by BODIPY™ 493/503 staining (Figure 3A). At the transcriptional level, differentiated FAPs showed decreased expression of the FAP-associated marker *PDGFRA*; however, detectable amplification was obtained in only one of three independent batches. In contrast, expression of the adipocyte-associated gene *CIDEC* increased across all three batches in both BNS1- and BNS2-derived FAPs (Figures 3B and C).

Expression of *FABP4*, a marker associated with mature adipocyte lipid metabolism [33], increased in BNS2-derived FAPs but remained undetectable in BNS1-derived FAPs following adipogenic induction. BNS1-derived FAPs also showed comparatively lower lipid-droplet accumulation than BNS2-derived FAPs. Together, these findings demonstrated isolate-dependent differences in adipogenic differentiation-associated responses.

**Figure 3:**
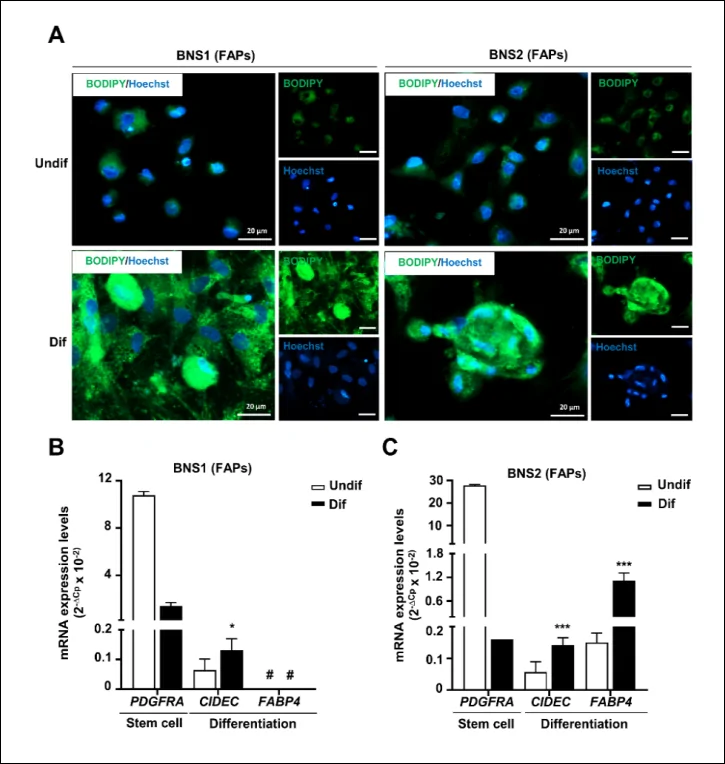
Adipogenic differentiation capacity of CD29⁺/CD56⁻ fibro-adipogenic progenitor cells. (A) Lipid-droplet accumulation in FAPs visualized using BODIPY™ 493/503 staining (green) after 14 days of culture in ADM; nuclei were counterstained with Hoechst 33342 (blue). Representative images were captured at 40× objective magnification. Scale bar = 20 µm. Messenger RNA expression of the FAP-associated marker *PDGFRA* and adipocyte-associated genes *CIDEC* and *FABP4* in (B) BNS1-derived and (C) BNS2-derived FAPs. Undif = Undifferentiated FAPs maintained in 15% FBS-MEM-α for 14 days; Dif = FAPs cultured in ADM for 14 days. Data are presented as mean ± standard deviation from three independent batches (n = 3). For *PDGFRA*, detectable amplification was obtained in one of three independent batches for both BNS1- and BNS2-derived FAPs (n = 1). *p* < *0.05; ***p* < *0.001, Student's t-test. #Undetectable. ADM = Adipogenic differentiation medium; FAPs = Fibro-adipogenic progenitor cells; FBS = Fetal bovine serum; MEM-α = Minimum essential medium alpha.

**Myogenic differentiation of SCs:** SCs derived from BNS2 and BNS3 were cultured in MDM on collagen type I-coated plates for 7 days. Following myogenic induction, the cells formed elongated, multinucleated myotube-like structures with strong MyHC immunoreactivity compared with undifferentiated controls (Figure 4A). Gene expression analysis showed that the myogenic differentiation-associated genes *Myf5*, *MyHC*, and *MyoG* were undetectable in undifferentiated SCs but became detectable following myogenic induction. Conversely, the SC-associated genes *Pax3* and *Pax7* were detected only in undifferentiated SCs (Figures 4B and C).

*Pax7* expression was consistently detected across the evaluated SC isolates, whereas *Pax3* expression varied between isolates. *Pax3* was detected in all three independent RNA batches from BNS2 but in only one of three batches from BNS3, consistent with previously reported inter-isolate variability in bovine MDSCs [6, 9, 29].

**Figure 4:**
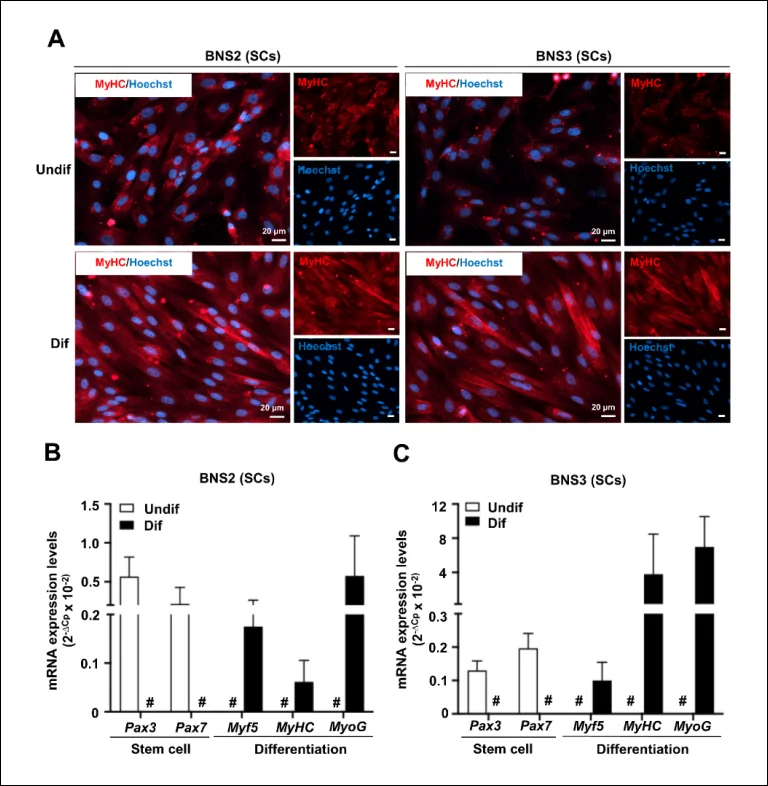
Myogenic differentiation capacity of CD29⁺/CD56⁺ satellite cells. (A) Immunofluorescence detection of MyHC (red) in SCs after 7 days of culture in MDM on collagen type I-coated plates; nuclei were counterstained with Hoechst 33342 (blue). Representative images were captured at 20× objective magnification. Scale bar = 20 µm. Messenger RNA expression of the SC-associated genes *Pax3* and *Pax7* and myogenic differentiation-associated genes *Myf5*, *MyHC*, and *MyoG* in (B) BNS2-derived and (C) BNS3-derived SCs. Undif = SCs maintained in BMCM for 7 days; Dif = SCs cultured on collagen type I-coated plates in MDM for 7 days. In both BNS2- and BNS3-derived SCs, each marker was detectable in one culture condition and undetectable in the other; therefore, no comparison contained quantitative values for both conditions, and statistical testing was not applicable. #Undetectable. BMCM = Basic muscle-derived stem cell culture medium; MDM = Myogenic differentiation medium; MyHC = Myosin heavy chain; SCs = Satellite cells.

**Lineage-committed subpopulations drive differentiation responses in heterogeneous CD29-positive MDSCs**

To determine the lineage-specific differentiation capacity of unfractionated CD29-positive populations, BNS2 cells were cultured under myogenic or adipogenic induction conditions without prior FACS-based separation of the constituent subpopulations (Figure 5A). Under adipogenic induction conditions, the cells exhibited prominent lipid accumulation with minimal MyHC immunoreactivity (Figure 5B). Conversely, under MDM-2 conditions, MyHC immunoreactivity increased markedly while lipid accumulation was suppressed, irrespective of whether the culture surface was collagen-coated (Figure 5C). Similar condition-dependent responses were reproduced using cryopreserved BNS2 cells.

These reciprocal differentiation responses demonstrated that heterogeneous CD29-positive MDSC populations contained cells capable of responding selectively to myogenic and adipogenic induction conditions. In conjunction with the FACS-defined CD56-positive SC and CD56-negative FAP subpopulations, these findings support an association between subpopulation composition and the lineage-specific differentiation behavior of unfractionated CD29-positive MDSCs [9, 25].

**Figure 5:**
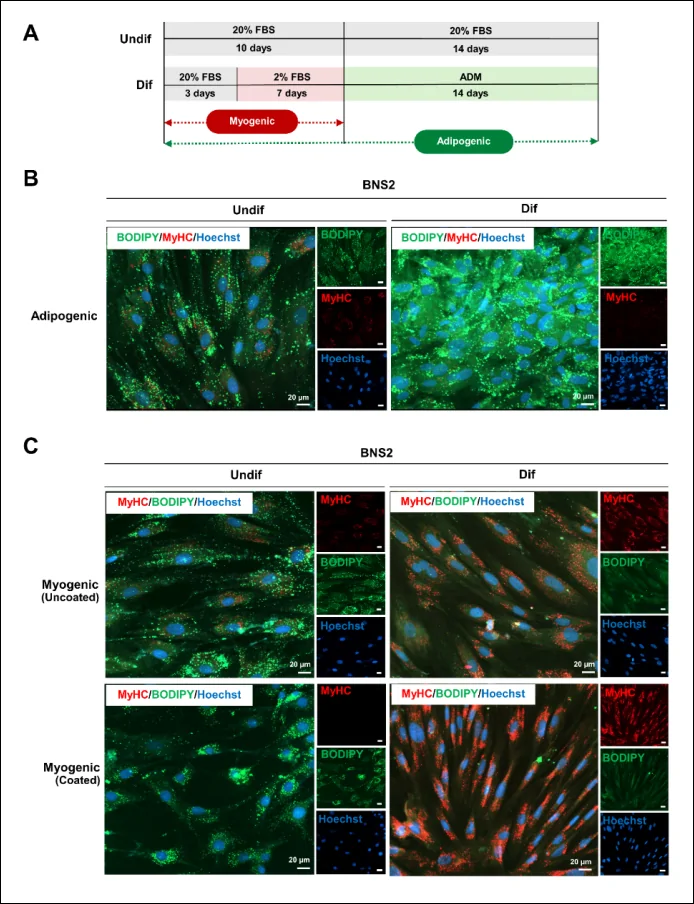
Lineage-specific differentiation responses of unfractionated CD29-positive muscle-derived stem cells. (A) Schematic representation of the sequential induction protocol used to direct unfractionated BNS2 MDSCs toward adipogenic or myogenic differentiation. (B) Adipogenic differentiation assessed by BODIPY™ 493/503 staining (green) following 14 days of ADM culture; nuclei were counterstained with Hoechst 33342 (blue). Representative images were captured at 20× objective magnification. Scale bar = 20 µm. (C) Myogenic differentiation assessed by MyHC immunofluorescence (red) following 7 days of culture in MDM-2 on collagen-coated or uncoated plates; nuclei were counterstained with Hoechst 33342 (blue). Scale bar = 20 µm. Undif = Unfractionated BNS2 MDSCs maintained in BMCM; Dif (adipogenic) = Cells cultured in BMCM for 3 days, followed by MDM-2 for 7 days and ADM for 14 days; Dif (myogenic) = Cells cultured in BMCM for 3 days followed by MDM-2 for 7 days on collagen-coated or uncoated plates. ADM = Adipogenic differentiation medium; BMCM = Basic muscle-derived stem cell culture medium; MDM-2 = Myogenic differentiation medium-2; MDSCs = Muscle-derived stem cells; MyHC = Myosin heavy chain.

## DISCUSSION

### Principal findings and biological interpretation

This study provides evidence that bovine CD29-positive MDSCs are better interpreted as heterogeneous mixtures of lineage-biased progenitor subpopulations than as a uniformly bipotent progenitor pool. This interpretation is supported by the marked inter-isolate variation in the relative proportions of SCs and FAPs and by the distinct lineage-specific responses observed after myogenic and adipogenic induction. Accordingly, the apparent dual-lineage differentiation capacity of unfractionated CD29-positive populations may be explained, at least in part, by their spatially variable cellular composition rather than by intrinsic multipotency of individual cells.

In addition to this biological finding, the present study established a reproducible and field-compatible protocol for isolating CD29-positive MDSCs from adult Pon Yang Kham Thai-French crossbred cattle. The protocol yielded 5.13 ± 1.23 × 10⁵ viable cells/g of tissue, achieved >95% CD29-positive enrichment after pre-sorting expansion, and supported robust proliferation during early passages. The capacity to recover and expand progenitor cells from adult slaughterhouse-derived skeletal muscle is relevant to veterinary biotechnology and cultured meat development, where accessible and standardized cell sources are required.

### Spatial heterogeneity of SC and FAP subpopulations

A major finding of this study was the pronounced variation in SC:FAP composition among spatial isolates. Individual isolates ranged from FAP-dominant to SC-enriched populations, indicating substantial heterogeneity within the sampled chuck roll tissue. SCs and FAPs have distinct but complementary biological roles, with SCs contributing primarily to myogenesis and FAPs contributing to adipogenic and stromal processes [26, 34, 35]. In livestock, these functions are particularly relevant because muscle growth and intramuscular fat deposition contribute to important meat quality characteristics, including marbling [26, 34, 35].

The observed variation may reflect differences in local tissue composition and microenvironment. Previous studies show that muscle stem cells and progenitor populations can vary by age, tissue context, and muscle characteristics [26, 34, 35]. However, the present experimental design does not allow separation of the relative contributions of genetic background, age, nutritional status, or the local muscle microenvironment. In addition, because the chuck roll was not seam-dissected and the individual muscle of origin of each isolate was not recorded, intermuscular variation may also have contributed to the observed SC:FAP differences.

Mechanistically, pathways such as Wnt and transforming growth factor-β (TGF-β) regulate myogenic and adipogenic lineage behavior in experimental systems [36, 37]. Whether comparable signaling mechanisms contribute to spatial variation in bovine SC and FAP abundance remains uncertain. Future studies should investigate whether lineage bias in adult bovine muscle is associated with stable molecular or epigenetic differences or remains responsive to extrinsic cues during *in vitro* expansion.

### Practical performance of the isolation protocol

The present protocol prioritizes simplicity, accessibility, and compatibility with adult slaughterhouse-derived tissue. Compared with previously reported approaches, the workflow incorporated short-term culture enrichment before phenotypic characterization and FACS, thereby achieving high CD29-positive enrichment without density-gradient centrifugation [9]. Differences in reported cell yield among studies should nevertheless be interpreted cautiously because donor age, tissue source, digestion conditions, and enrichment strategy can substantially influence recovery. Adult skeletal muscle may also contain fewer expandable progenitor cells than fetal or juvenile tissue [26, 34, 35].

Supplementary Table 3 provides a direct comparison with representative bovine MDSC isolation methods, including those described by Naraoka *et al.* [9] and Dohmen *et al.* [6]. The present protocol yielded 5.13 ± 1.23 × 10⁵ viable cells/g of tissue and achieved >95% CD29-positive enrichment using skeletal muscle obtained from market-weight adult cattle aged 2.5–3 years. In contrast, several previously reported protocols have relied on fetal or juvenile tissues, which may not reflect the practical cell sources available through commercial slaughter systems. The present workflow also used single-step enzymatic digestion and did not require density-gradient centrifugation. Furthermore, the successful recovery of viable cells from cryopreserved tissue supports the potential use of tissue banking for subsequent experiments, although cryopreservation stability beyond 100 days was not evaluated.

### Proliferative capacity and p38 MAPK inhibition

Both SCs and FAPs showed strong proliferative capacity during early passages, followed by a progressive decline at later passages. This pattern is consistent with the finite proliferative lifespan commonly observed in primary adult muscle-derived progenitor cells [30–32]. The gradual increase in population doubling time across passages further supports the importance of limiting prolonged expansion when cells are intended for downstream differentiation experiments.

The culture medium contained 2.5 µM of the p38 MAPK inhibitor SB203580, a lower concentration than that used in several previous studies [25, 29, 30, 38]. Under the conditions used here, cells retained both proliferative and lineage-specific differentiation capacity. These findings indicate that 2.5 µM SB203580 was compatible with the present culture system. However, because the study did not directly compare different SB203580 concen-trations, it cannot establish that this concentration is superior, more cost-effective, or biologically optimal. Direct dose-response experiments are needed to determine the concentration that best balances expansion and maintenance of differentiation potential.

### Lineage-specific differentiation of FAPs and SCs

The differentiation-associated responses of isolated FAPs and SCs further supported their functional distinction. FAPs from BNS1 and BNS2 accumulated intracellular lipids after adipogenic induction and showed increased *CIDEC* expression. In BNS2-derived FAPs, *FABP4* expression also increased, whereas *FABP4* remained undetectable in BNS1-derived FAPs despite lipid accumulation. Because *FABP4* is associated with adipocyte differentiation and lipid metabolism [33, 39, 40], this isolate-dependent difference may indicate variation in the extent of adipogenic differentiation. However, the present data do not establish a definitive temporal sequence between *CIDEC* and *FABP4* expression. The interpretation that these markers represent different stages of adipogenic progression is biologically plausible and consistent with previous studies [41, 42], but it should be validated with time-course experiments and additional adipogenic markers.

SCs derived from BNS2 and BNS3 formed multinucleated myotube-like structures and showed MyHC immunoreactivity following myogenic induction. The myogenic differentiation-associated genes *Myf5*, *MyHC*, and *MyoG* became detectable after induction, whereas the SC-associated genes *Pax3* and *Pax7* were detected in undifferentiated cells. *Pax7* was detected more consistently than *Pax3* across the evaluated SC isolates. Previous studies have also described variability in satellite cell-associated marker expression in bovine muscle-derived populations [6, 9, 25, 29]. These findings further emphasize that characterization of individual isolates may be more informative than assuming uniform marker expression across all bovine MDSC populations.

### Interpretation of apparent CD29-positive MDSC bipotency

The reciprocal lineage responses observed in unfractionated CD29-positive populations provide important context for interpreting their apparent bipotency. Under adipogenic induction, the cells showed prominent lipid accumulation with minimal MyHC expression, whereas myogenic induction increased MyHC expression and suppressed lipid accumulation. In parallel, FACS identified distinct CD29⁺/CD56⁺ SC and CD29⁺/CD56⁻ FAP subpopulations within the bulk CD29-positive fraction.

Taken together, these findings support a model in which the differentiation behavior of bulk CD29-positive MDSCs is influenced by the relative abundance and selective response of pre-existing lineage-biased subpopulations. This interpretation is consistent with single-cell transcriptomic evidence showing that bovine SCs and FAPs constitute transcriptionally distinct populations before *in vitro* manipulation [31]. However, the present study did not perform single-cell lineage tracing; therefore, intrinsic bipotency at the level of individual CD29-positive cells cannot be completely excluded. The findings instead demonstrate that population level dual-lineage differentiation alone is insufficient to establish intrinsic single-cell multipotency.

### Implications for cultured meat production

The spatial variation in SC:FAP composition has practical implications for cultured meat production because uncontrolled differences in starting-cell composition may affect subsequent biomass expansion, myogenic differentiation, adipogenic differentiation, and tissue composition. If bulk MDSC preparations differ substantially in their baseline SC:FAP ratios, downstream responses may vary even when the same culture and induction conditions are applied.

Accordingly, characterization of subpopulation composition before large-scale expansion may improve process reproducibility. Potential strategies include prospective enrichment, standardized SC:FAP profiling, or culture conditions designed to accommodate defined mixtures of lineage-committed progenitors. However, the present study did not directly evaluate large-scale manufacturing performance, batch-to-batch variability across independent donors, or final cultured meat characteristics. Therefore, the suggestion that baseline SC:FAP composition may contribute to inconsistencies reported in cultured meat production should be considered a testable hypothesis rather than a demonstrated causal mechanism.

The present findings also have potential implications for donor selection and cell-bank development. Rapid assessment of SC:FAP composition before large-scale culture could help identify tissue sources with predictable myogenic or adipogenic potential. Genetic or epigenetic screening may also be useful in this context, but the present study did not evaluate these approaches, and they require independent validation before they can be recommended for routine cell sourcing.

### Industrial translation and process standardization

Successful translation of cultured meat technologies will require scalable cell expansion platforms, serum-free or chemically defined culture systems, standardized upstream processing, and careful control of manufacturing costs. Although the present workflow provides a practical method for isolating cells from adult slaughterhouse-derived tissue, its compatibility with large-scale bioreactor expansion and food-grade production remains to be established.

Subpopulation heterogeneity may represent both a biological and a process-control variable. Variation in SC:FAP ratios could potentially influence biomass yield, final tissue composition, and differentiation performance. Consequently, subpopulation-aware quality control may be useful during process development. Nevertheless, this study did not directly examine the effects of SC:FAP composition on media consumption, manufacturing cost, bioreactor performance, or final product quality; future scale-up studies should evaluate these effects experimentally.

### Study limitations and future directions

Several limitations should be considered when interpreting these findings. First, detailed subpopulation characterization and functional analyses were based on three spatial isolates obtained from a single donor animal. Therefore, the observed SC:FAP ratios cannot be generalized to the broader Pon Yang Kham Thai-French crossbred cattle population. Although the within-donor design was useful for demonstrating spatial heterogeneity, larger studies involving genetically and phenotypically diverse animals are required to quantify inter-animal variability and establish population level distributions of SC:FAP composition.

Second, although BNS1, BNS2, and BNS3 were obtained from distinct anatomical positions within the chuck roll, the subprimal was not seam-dissected and the specific muscle of origin of each isolate was not recorded. Because SC and FAP abundance may vary with muscle characteristics and fiber-type composition, intermuscular differences may have contributed to the observed heterogeneity. Future studies should therefore use individually identified muscles and standardized anatomical sampling locations.

Third, the culture system relied on FBS, which limits direct translation to food-grade cultured meat production because of ethical, regulatory, reproducibility, and scalability considerations [13, 23, 43–45]. Development and validation of serum-free or chemically defined media will therefore be necessary for subsequent translational applications.

Fourth, the findings require validation in larger and more diverse donor populations before breed-level conclusions can be drawn. Longitudinal studies should also evaluate whether SC:FAP composition remains stable during expansion and whether donor age, sex, genotype, nutritional status, or muscle type systematically influences subpopulation abundance.

Fifth, the gene expression panel was designed primarily for lineage identification and did not include key upstream regulators such as *PPARG* for adipogenic commitment or *MYOD1* for early myogenic activation [46, 47]. The authors also did not perform protein-level validation by Western blotting, enzyme-linked immunosorbent assay, or other quantitative approaches. In addition, quantitative assessment of myotube fusion, lipid-droplet accumulation, and contractile function would strengthen functional characterization. Future studies should therefore incorporate broader transcriptomic or proteomic profiling, validated reference genes, fusion-index analysis, quantitative lipid measurements, and functional contractility assays.

Sixth, the *PDGFRA* findings shown in Figure 3 were based on detectable amplification in only one of three independent RNA batches for both BNS1- and BNS2-derived FAPs. In that batch, *PDGFRA* was abundantly detected, whereas amplification was absent in the remaining two runs. The >11-Cp difference among runs, together with the identical failure pattern in two independently derived FAP isolates analyzed within the same runs, suggests that technical assay failure may have contributed to the missing amplification [48]. Because the remaining cDNA was exhausted, the failed runs could not be repeated. Consequently, the apparent reduction in *PDGFRA* expression after adipogenic induction should be considered preliminary and requires independent confirmation.

Seventh, warm ischemia time between slaughter and tissue processing was not formally recorded. This factor may affect cell viability, recovery, and subsequent culture performance, and documenting it would improve reproducibility between laboratories [49]. Future studies should therefore standardize and report the interval between slaughter, tissue collection, cooling, and processing.

Overall, future work should prioritize multi-donor validation, standardized anatomical sampling, serum-free culture systems, broader molecular and functional characterization, long-term cryopreservation studies, and scale-up experiments using controlled SC:FAP compositions. These approaches will be important for determining whether subpopulation-aware cell sourcing can improve the reproducibility and industrial feasibility of bovine cultured meat production.

## CONCLUSION

This study established a reproducible and field-compatible approach for isolating and expanding CD29-positive MDSCs from adult Pon Yang Kham Thai-French crossbred cattle. The protocol yielded 5.13 ± 1.23 × 10⁵ viable cells/g of tissue, achieved >95% CD29-positive enrichment, and supported robust early-passage proliferation. Importantly, the CD29-positive populations showed pronounced spatial heterogeneity in SC:FAP composition, ranging from 4.3:95.1 in the FAP-dominant BNS1 isolate to 96.0:3.8 in the SC-enriched BNS3 isolate. Lineage-specific assays further showed that CD56-positive SCs formed multinucleated, myotube-like structures with MyHC immunoreactivity and expression of myogenic differentiation-associated genes, whereas CD56-negative FAPs accumulated intracellular lipids and showed expression of adipogenic differentiation-associated genes. The reciprocal responses of unfractionated CD29-positive populations under myogenic and adipogenic induction conditions indicate that their apparent dual-lineage differentiation is strongly influenced by the relative composition and selective response of pre-existing lineage-biased subpopulations.

From a practical perspective, these findings highlight the importance of considering SC:FAP composition as a potential source of variability during bovine cultured meat production. Reliance on unfractionated MDSC populations without prior characterization may contribute to inconsistent differentiation outcomes and tissue composition. Therefore, subpopulation-aware cell sourcing, prospective enrichment, and routine assessment of SC:FAP ratios may improve reproducibility during cell expansion and subsequent tissue engineering. The use of adult slaughterhouse-derived skeletal muscle and the demonstrated feasibility of tissue cryopreservation also strengthen the practical relevance of the protocol for livestock biotechnology and future cell-banking applications.

A major strength of the study is the integration of a simplified isolation workflow with phenotypic characterization, FACS-based subpopulation separation, proliferation assessment, lineage-specific differentiation, gene expression analysis, and evaluation of unfractionated CD29-positive populations. This combined approach allowed the cellular composition of the bulk population to be linked directly with functional differentiation behavior rather than inferring bipotency from population level assays alone.

Overall, the findings support a revised interpretation of bovine CD29-positive MDSCs as spatially heterogeneous mixtures of lineage-biased SC and FAP subpopulations rather than a uniformly bipotent cell pool. Accounting for this heterogeneity will be important for improving experimental reproducibility and developing standardized cell sources for cultured meat applications. Future work should validate these findings across larger and more diverse donor populations and determine how controlled SC:FAP compositions influence scalable expansion, structured muscle-fat tissue formation, and final product consistency.

## DATA AVAILABILITY

 The supplementary data can be obtained from the corresponding author upon reasonable request.

## AUTHORS’ CONTRIBUTIONS

KV: Conceptualization, methodology, investigation, formal analysis, validation, data curation, writing – original draft, writing – review and editing, visualization, supervision, and project administration. SS: Methodology, investigation, formal analysis, visualization, validation, data curation, and writing – original draft. NS: Methodology, investigation, and visualization. JP: Methodology, investigation, and visualization. RT: Conceptualization and methodology. VL and KP: Conceptualization, supervision, and revised the manuscript. RP, AC, and TB: Conceptualization and supervision. All authors have read and approved the final version of the manuscript.
